# Employees and the National Insurance Act

**Published:** 1912-09-14

**Authors:** 


### NATIONAL INSURANCE ACT DEPARTMENT.

### EMPLOYEES AND THE NATIONAL INSURANCE ACT.

### XXVII.—Interpretation of the Act.

During the past few weeks several cases arising out of the National Insurance Act have come before the Courts for adjudication, and the decisions are of considerable importance. The cases have been of two kinds—namely: (1) those in which the Insurance Commissioners have taken advantage of the provisions of Section 66 for the determination of questions under the Act, and (2) those in the nature of summonses for offences against the Act. We propose in this article to consider in the first place the provisions of the Act regulating the procedure in these cases, and then to furnish a brief summary of the decisions in the particular cases alluded to.

Determination of Questions.

The Act expressly provides under Section 66 the classes of questions that the Insurance Commissioners are empowered to decide, and further sets forth the procedure to be adopted, in the event of any person feeling aggrieved by their decision, for obtaining the adjudication of the Court. The classes of questions that may be so determined by the Commissioners are: (1) whether any employment or class of employment is such as to bring the employed person within the description of those who have to be insured, or as to whether a person is entitled to become a voluntary contributor; (2) those dealing with the rate of contribution; and (3) those dealing with the apportionment of the contribution between the employer and employee respectively.

Individual questions arising under either of these groups are to be determined in the first instance by the Insurance Commissioners in accordance with regulations made by them for the purpose, but they are empowered, if they think fit, instead of themselves deciding whether a particular employment is such as to render the person following it subject to the compulsory provisions of the Act, to submit the question for decision to the High Court. Applications of this description have to be made in accordance with the rules of the Court, and after hearing the parties and taking such evidence as it thinks just, the Court is to decide the question and its decision is final.

It is under this special provision that the cases, presently mentioned, have been brought before the Courts. This is in itself sufficient indication of the importance of the questions involved, because it shows that the Insurance Commissioners, although invested with exceedingly wide powers, were unable to interpret the Act in its detailed application in the particular instances without the assistance of the Court.

If the Insurance Commissioners themselves decide a question falling within class (1) as above described, and any person feels aggrieved by that decision, it is open to him to appeal therefrom to the County Court. The decision of the County Court as to the facts of the case is apparently final, but there is a further right of appeal on any question of law to such judge of the High Court as may be appointed by the Lord Chancellor, and the decision of that judge is final.

Employment Within the Meaning of the Act. Contract of Employment.

To be compulsorily insured under the Act a person must be in employment under contract of service (First Schedule, Part I. (*a*)). The term "Contract of Service" is not defined in the Act, though it is amplified by the statement that it may be either written or oral, and may be in express terms or merely implied. Two decisions have been given by the Courts bearing on the question of the contract of service, and, although it is likely that others may have to be submitted to adjudication, these decisions have made the position quite clear as to compulsory insurance in the two classes of occupation specifically brought to the notice of the Court.

The first decision was that of Mr. Justice Joyce in the Chancery Division of the High Court on July 11 last, when two cases of a similar character were heard. Both cases referred to ministers of the United Methodist Church, and they were differentiated from each other merely on the ground that in the first instance duly appointed ministers were concerned, whilst in the second case the question referred to ministers on probation. From the arguments of counsel it appeared that the Conference of the Church has power under its Deed Poll to appoint ministers and probationers, and to dismiss the latter if not satisfied with their general suitability. The Conference also has similar powers of suspending ministers upon satisfactory evidence of neglect of duty or of general unsuitability. The Conference does not, however, pay the ministers for their services, this being arranged by the particular circuit to which they are attached. It was contended on these grounds that the control exercised by the Conference was only partial and that there was a distinction between a "contract of service" and a "contract for service." The services of the ministers appeared to come under the latter category, for although control is exercisable by the Conference so far as the power of suspension is concerned, the ministers have complete independence in the detailed conduct of their work. In delivering judgment Mr. Justice Joyce reviewed these arguments, and said that if the persons affected were employed within the meaning of the Act it would be a difficult matter to decide who was responsible for the payment of the contributions, and that in his opinion there was no contract of service in either case.

In so far as it is possible to impart a general application to this finding it would seem that it is essential in a contract of service that the employer should have the power of controlling the manner in which the service is rendered as well as the power of dismissing the servant.

### Two More Cases.

Two similar cases were brought before Mr. Justice Parker on July 26. Both cases referred to curates of the Church of England, and the distinction between them was again the difference between a fully licensed curate and one on probation holding a temporary licence. The question for decision was whether the curates were compulsorily insurable under the Act, and in his judgment Mr. Justice Parker made it clear that as, in his opinion, there did not exist any contract of service between the curates and their vicars or any other person, they were not employed within the meaning of the Act. His Lordship pointed out that, although a curate must obey his vicar, the latter has not the power either to employ or to dismiss from his services a curate except on the authority of a licence or consent of his Bishop. Such services as were rendered were in the nature of assistance to an ecclesiastical superior and the curate was not bound under contract. It would thus seem that the general application of the finding amplifies that of the case of the Wesleyan ministers by making it necessary that there shall be power to employ and to dismiss from employment as well as power of control of the services rendered, in order that there may be a contract of service.

### Manual Labour.

Under the First Schedule to the Act, Part II. (*g*), employment otherwise than by way of manual labour and at a rate of remuneration exceeding in value £160 a year is excepted from compulsory insurance. No definition is furnished in the Act of the term "manual labour," and an application was made to Mr. Justice Swinfen Eady on July 30 last under the provisions of Section 66, above referred to, to decide whether the employment of tailors' cutters and dairymen's foremen came within the meaning of the term. Individual cases were presented in order to obtain a complete statement of the facts. The tailor's cutter held the view that he was outside the operation of the Act, whilst the dairymen's foreman wished to be insured. It should be mentioned that in both cases the rate of remuneration exceeded £160 a year. In delivering judgment, Mr. Justice Swinfen Eady reviewed the findings as to the meaning of the term "manual labour" as decided in cases under the Employers' and Workmen's Compensation Acts. In particular he quoted the decision of Lord Justice Fry, with reference to a grocer's assistant, as follows: "If the words 'manual labour' are to have the full significance which could be put on them they would be extended to every kind of employment. That cannot be the true meaning of the statute, but some more confined interpretation must be arrived at. I agree that this must be done by looking to the nature of the substantial employment, and not to matters that are incidental and accessory. The determination of what is substantial and what accessory may be a question of difficulty, but in my view of the case the appellant (the grocer's assistant) was not engaged in manual labour."

On reviewing the facts of the case of the dairymen's foreman, Mr. Justice Swinfen Eady came to the conclusion that the substantial employment was that of a branch manager, and that the lifting of churns was merely accessory, and in consequence he was not engaged in manual labour. The case was clearer still in regard to the tailor's cutter, for, although there might be manual work in actually cutting out the cloth, the substantial employment was that of a branch manager, and that in the matter of designing clothes a considerable amount of technical skill was requisite.

It was, therefore, held in both cases that the employees were not engaged in manual labour, and did not have to be insured. No general principles appear to have been decided in these cases, and it is still left, so far as we can see, to the consideration of the particular facts of individual cases before any definite decision can be given.

### Offences against the Act.

The other class of cases to which we referred at the commencement of this article is that of the prosecution of employers for having failed to carry out their duties in regard to stamping the Insurance cards of certain of their employees. Proceedings of this character are governed by Section 69, which deals with offences, and being of a summary nature are heard in the Police Courts. It is unnecessary for us to enter into a detailed description of the cases, but it may be said that the main contention of the employers, as represented by the Insurance Tax Resistance Society, was that the Act was in the nature of a contract of insurance, and that, as arrangements have not yet been completed for administering medical and sanatorium benefits, there was no liability to pay the contributions.

In giving his decision in the first of these cases, Mr. Baggallay, at the Lambeth Police Court on August 20, said he could not take into account whether or not the necessary arrangements had been made for the administration of the benefits under the Act, but that, reviewing the evidence, he came to the conclusion that the omission to stamp the cards was deliberate and not merely a matter of convenience, and in consequence an offence had been committed against the Act. Three summonses in respect of three employees were dealt with, and on each a penalty of £5 was imposed, with £5 5s. costs on the first one. Penalties of a similar onerous character have been imposed in the other cases that have since been heard.

In addition, it was ordered that, in conformity with Section 69, the contributions should be paid. It is to be noted that the penalty, large though it is, is not the maximum amount prescribed by the Act. The fine may be as much as £10 for each offence.

---

**NOTE.—Questions and Correspondence on all doubtful points are invited, and should be addressed to the Editor, The Hospital, 28 Southampton Street, Strand, W.C., and marked "Insurance."**

